# Neuregulin 1-Beta Cytoprotective Role in AML 12 Mouse Hepatocytes Exposed to Pentachlorophenol

**DOI:** 10.3390/ijerph2006030002

**Published:** 2006-03-31

**Authors:** Waneene C. Dorsey, Paul B. Tchounwou, Byron D. Ford

**Affiliations:** 1 Molecular Toxicology Research Laboratory, Grambling State University, Grambling, LA, USA.; 2 Molecular Toxicology Research Laboratory, NIH-Center for Environmental Health, College of Science, Engineering, and Technology, Jackson State University, Jackson, MS, USA.; 3 Department of Anatomy and Neurobiology, Morehouse School of Medicine, Atlanta, GA, USA

**Keywords:** Cytoprotection, Neuregulin1-ß, Pentachlorophenol, Cytotoxicity, Mouse Hepatocytes

## Abstract

Neuregulins are a family of growth factor domain proteins that are structurally related to the epidermal growth factor. Accumulating evidence has shown that neuregulins have cyto- and neuroprotective properties in various cell types. In particular, the neuregulin-1 βeta (NRG1-β) isoform is well documented for its anti-inflammatory properties in rat brain after acute stroke episodes. Pentachlorophenol (PCP) is an organochlorine compound that has been widely used as a biocide in several industrial, agricultural, and domestic applications. Previous investigations from our laboratory have demonstrated that PCP exerts both cytotoxic and mitogenic effects in human liver carcinoma (HepG_2_) cells, primary catfish hepatocytes and AML 12 mouse hepatocytes. We have also shown that in HepG_2_ cells, PCP has the ability to induce stress genes that may play a role in the molecular events leading to toxicity and tumorigenesis. In the present study, we hypothesize that NRG1-β will exert its cytoprotective effects in PCP-treated AML 12 mouse hepatocytes by its ability to suppress the toxic effects of PCP. To test this hypothesis, we performed the MTT-cell respiration assay to assess cell viability, and Western-blot analysis to assess stress-related proteins as a consequence of PCP exposure. Data obtained from 48 h-viability studies demonstrated a biphasic response; showing a dose-dependent increase in cell viability within the range of 0 to 3.87 μg/mL, and a gradual decrease within the concentration range of 7.75 to 31.0 μg/mL in concomitant treatments of NRG1-β+PCP and PCP. Cell viability percentages indicated that NRG1-β+PCP-treated cells were not significantly impaired, while PCP-treated cells were appreciably affected; suggesting that NRG1-β has the ability to suppress the toxic effects of PCP. Western Blot analysis demonstrated the potential of PCP to induce oxidative stress and inflammatory response (*c-fos*), growth arrest and DNA damage (GADD153), proteotoxic effects (HSP70), cell cycle arrest as consequence of DNA damage (p53), mitogenic response (cyclin-D1), and apoptosis (caspase-3). NRG1-β exposure attenuated stress-related protein expression in PCP-treated AML 12 mouse hepatocytes. Here we provide clear evidence that NRG1-β exerts cytoprotective effects in AML 12 mouse hepatocytes exposed to PCP.

## Introduction

Neuregulins are transmembrane polypeptide growth factors with structural epidermal growth factor (EGF)-like domains. The neuregulin EGF-like domain binds with four estrogene receptor B (ErbB receptor)-tyrosine kinases, thereby orchestrating a growth factor signaling system essential for cell growth, differentiation, and survival [1–3]. It is well documented that neuregulin interaction with ErbB receptors can result in receptor dimerization, tyrosine phosphorylation, and subsequent activation of intracellular signaling pathways [2, 4]. Neuregulins are highly expressed in the nervous system, where they play crucial roles in development, maintenance, and repair [5]. Moreover, neuronal migration, synaptogenesis, receptor subunit composition, and the proliferation and survival of Schwann cells, and oligodendrocytes are influenced by neuregulin-ErbB receptor interaction [5, 6]. For example, neuregulin diminishes autoimmune demyelination, promotes oligodendrocyte progenitor expansion, and enhances remyelination in the central nervous system [7].

Neuregulins (NRG) are synthesized from alternative spliced transcripts of one of four known (neuregulin-1,-2, -3, and -4) genes. To date, neuregulin-1 (NRG1) gene transcripts encoding over 15 different isoforms have been identified [8]. NRG1 is also called *neu* differentiation factor, heregulin, or glial growth factor, and has acetylcholine-receptor-inducing activity. More specifically, the NRG1 gene expresses a α- or β-type EGF-like domain that preferentially binds to erbB3 and erbB4 tyrosine kinase receptors [1, 9, 10]. The NRG1-β isoform is predominant in the central nervous system and has been shown to participate in development, survival, and metabolism in neuron and glial cells [6, 11].

Accumulating evidence has shown that exogenous NRG1-β treatment can exert cyto- and neuroprotective effects in neuronal cells [4, 12, 13]. In addition, NRG1-β treatment prevents macrophage and microglial infiltration and astrocytic activation following focal ischemic stroke in the rat [12]. The same study also reported that the neuroprotective activity of NRG1-β can suppress interleukin-1 beta mRNA levels [12]. It has been demonstrated that NRG1-β blocks the induction of pro-inflammatory and stress genes provoked by ischemia [13]. In the presence of NRG1-β, Schwann cells infected with the N70-Ets DNA plasmid show minimum cell death [4]. The ability of NRG1-β to protect myocytes against anthracyline- and β-adrenergic receptor-induced cell injury and death is well documented [14–16].

Pentachlorophenol (PCP), an organochlorine fungicide, is a probable human carcinogen-Group B_2_ [17], based on suggestive evidence of carcinogenicity from laboratory animal studies. Previous findings from our laboratory have demonstrated that PCP has the ability to undergo Phase I biotransformation in the liver (CYP1A1 and XRE), to cause cell proliferation (*c-fos*), to cause growth arrest and DNA damage (GADD153 and p53), to influence the toxicokinetics of metal ions (HMTIIA), and to induce proteotoxic effects (HSP70 and GRP78) in HepG_2_ cells [18]. We have also reported that PCP exerts both cytotoxic and mitogenic effects in human liver carcinoma (HepG_2_) cells, primary catfish hepatocytes, and AML 12 mouse hepatocytes [19, 20]. In the present study, we hypothesized that NRG1-β will exert a cytoprotective effect in PCP-treated AML 12 mouse hepatocytes *in vitro*.

## Materials and Methods

### Chemicals

Pentachlorophenol (C_6_Cl_5_OH, CAS No. 87-86-5, Lot No. 01530TS), with purity 98.0% was purchased from Sigma-Aldrich Chem CO., (St. Louis, Missouri). Neuregulin 1-βeta (a gift from Dr. Byron Ford, Morehouse School of Medicine, Atlanta, GA; this is referred to as NRG1- β in the text). Dulbecco’s Modified Eagle’s Medium (DMEM) (Lot No. AQF24057) and Dulbecco’s phosphate buffered saline (PBS) (Lot No. AQE23425) was purchased from Hyclone (Logan, Utah). Tissue culture supplements were purchased from American Type Culture Collection (ATCC) Manassas, VA. Thiazolyl blue trazolium bromide CAS 298-93-1, purity 97.5%, β-mercaptoethanol, and dimethyl sulfoxide were purchased from Sigma-Aldrich (St. Louis, Missouri). Twelve percent SDS-PAGE gels were obtained from ISC BioExpress (Kaysville, UT). HSP70 primary monoclonal antibody was purchased from Calbiochem (La Jolla, CA). *c-fos*, caspase-3, cyclin D1, and p53, primary monoclonal antibodies, were purchased from Oncogene Research Products (San Diego, CA). The GADD153 primary polyclonal antibody was obtained from Abcam Inc. (Cambridge, MA). Alkaline phosphatase conjugated goat-anti-mouse IgG secondary antibody, and BCIP/NBT color development substrate were purchased from Promega (Madison, WI). Reagents for protein determination, gel electrophoresis, and Western blot analysis were obtained from Bio-Rad (Hercules, CA).

### Cell Culture

Alpha mouse liver 12 (AML 12) hepatocyte cultures were established from a mouse transgenic for human transforming growth factor α (ATCC CRL-2254, Manassas, VA). The cells were stored in liquid nitrogen until future use. The content of each vial was transferred to a 75 cm^2^ tissue culture flask diluted with DMEM, supplemented with 10% fetal bovine serum (FBS) and 1% streptomycin-penicillin, and incubated at 37°C under an atmosphere of 5% CO_2_ with humidified air to allow the cells to grow and form a monolayer in the flask. Subsequently, cells grown to 80–95% confluence were washed with PBS, trypsinized with 5 mL of 0.25% (w/v) EDTA, diluted, counted, and seeded in 96-well microtiter tissue culture plates (5 × 10^5^ cells/well) for cell viability studies.

### Cell Viability Experiments

To establish cell viability in hepatocytes treated with concomitant doses of NRG1-β+PCP and PCP. Administered doses ranged from 0 to 31.0 µg/mL for an exposure period of 48h. Prior to exposure, cells (5 × 10^5^) were maintained with medium containing 10% FBS. On the day of exposure, FBS-medium was removed and replaced with serum-free medium. In the concomitant experiments, a 0.01 nM NRG1-β was added to varying doses of PCP. Cell viability was evaluated using a colorimetric assay in which the reduction of a tetrazolium salt [3-(4,5-dimethylthiazol-2-yl)-2,5-diphenyltetrazolium bromide] (MTT) by the mitochondrial dehydrogenase of living cells was detected. In this assay, metabolically active cells were able to convert MTT to water-insoluble dark-blue formazan crystals. Viable cells were quantified by dissolution in 100% dimethyl sulfoxide and measured by absorbance with the wavelength set at 550 nm; using an EL 800 Model ELISA plate reader (Bio-Tek Instruments Inc., Winooski, Vermont) [21]. The toxic effect of PCP at different doses was expressed as the percentage of the absorbance determined for control cells incubated with the corresponding vehicle.

### Sample Collection and Protein Determination

Cells grown to 80–95% confluence were washed with PBS, trypsinized with 5 mL of 0.25% (w/v) EDTA, diluted, counted, and seeded in two 48-well microtiter tissue culture plates (1 × 10^6^ cells/well). Whole cells were treated with PCP and NRG1-β+PCP (0–16 μg/mL) for 48 h. Cells were resuspended in 300 μL of sample buffer (0.2 mol/L Tris, pH 6.8, 1% SDS, 30% glycerol, 7.5% β-mercaptoethanol, 0.1% bromophenol blue) per well. Cells were mechanically dislodged, transferred to microcentrifuge tubes, and heated at 95°C for 10 min. Samples were frozen until future use. The Bradford protein assay in a microtiter plate format was used for the determination of protein concentrations (20–25 μg) in samples. The total protein concentrations for cell lysates were quantitatively measured at 550 nm absorbance; using the Bio-Tek Model – EL 800 microplate reader.

### Western Blot Analysis for Identification of Specific Cellular Proteins

The Western-blot analysis was conducted to determine specific cellular response gene proteins (*c-fos*, caspase-3, cyclin D1, GADD153, HSP70, and p53) at 48 h of PCP and NRG1-β+PCP exposure to AML 12 mouse hepatocytes. Twenty micrograms of total protein from whole cells extracts were denatured in load buffer and separated using a 12% SDS–polyacramide gradient gel. After migration, gels were equilibrated in transfer buffer (20 mM Tris base, 150 mM glycine, 20% methanol, pH 8) and separated proteins were transferred onto a nitrocellulose membrane. Subsequently, the nitrocellulose membrane was blocked (10 mL Tris-buffered saline, 0.1 Tween-20 [TBST] with 5% nonfat dry milk) for 1 h at room temperature. Detection of membrane-bound proteins induced by PCP an d NRG1+PCP was carried out using specific primary antibodies that recognize proteins of interest (*c-fos* 15:1000, caspase-3 4:1000, cyclin D1 4:1000, GADD153 1:1000, HSP70 1:1000, and p53 1:1000). Subsequently, the reactions were reprobed with a secondary alkaline conjugated 1:7500 anti-mouse IgG antibody. BCIP/NBT color substrate was incorporated to develop protein bands. Immunoblot 1-D protein bands were assessed for relative abundance by TotalLab computer software (Nonlinear USA Inc. Durham, NC).

## Statistical Analysis

Absorbance readings of 550 nm from cell viability experiments were transformed into percentages to compare the viability of treated cells to that of untreated (control) cells. Graphs were made to illustrate the dose-response relationship with respect to cytotoxicity or cell viability. Standard deviations were determined, and the Student’s *t*-test values were computed to determine if there were significant differences in cell viability in PCP- and NRG1-β+PCP-treated cells compared to control cells (0, 0.01 nM NRG1-β). A value of *p*<0.05 was considered significant.

## Results

### Comparison of Untreated and NRG1-βTtreated Mouse Hepatocytes

**Figure 1:**
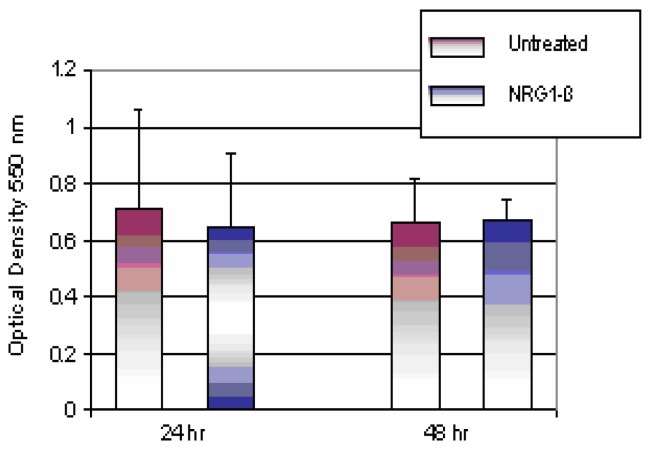
*Comparison of untreated and NRG1-β-treated AML 12 mouse hepatocytes*. NRG1-β-treated (10 nM NRG1-β; 1:1000) hepatocytes were compared to untreated (0) cells for 24- and 48 h. Hepatocytes were maintained in DMEM medium with 10% FBS supplement. On day of exposure, FBS-medium was replaced with serum-free medium. The MTT-assay was used to determine absorbance at 550 nm after 24- and 48 h exposure periods. Absorbance readings are expressed as optical density. Each bar represents the mean ± S.D (n=3 independent experiments; p>0.05).

The comparison of untreated and NRG1-β-treated mouse hepatocytes *in vitro* is shown in Figure 1. Neuregulin has the ability to elicit cell proliferation through interaction with members of the ErbB family of receptor tyrosine kinases [5, 6]. Therefore, it was of interest to determine whether NRG1-β treatment of hepatocyte culture *in vitro* could promote a mitogenic response. Cells were treated with 0.01 nM NRG1-β or left untreated (0) for 24- and 48 h periods according to the methodology section. The cells were assayed for viability by using MTT incorporation. The mean absorbance was recorded as optical density at 550 nm for untreated and NRG1-β treated cells. The optical densities for 24 h untreated (0) cells and 0.01 nM NRG1-β were recorded at 0.71 and 0.64, respectively. The 48 h optical density was recorded at 0.66 and 0.67 respectively for untreated (0) cells and 0.01 nM NRG1-β. Data obtained from these experiments demonstrated that differences in the mean absorbance of untreated and NRG1-β-treated cells were not statistically significant (*p*>0.05). Our findings suggest that AML 12 mouse hepatocytes do not significantly proliferate in response to NRG1-β treatment in serum-free medium.

### Effects of NRG1-β on PCP-Treated Mouse Hepatocytes

**Figure 2:**
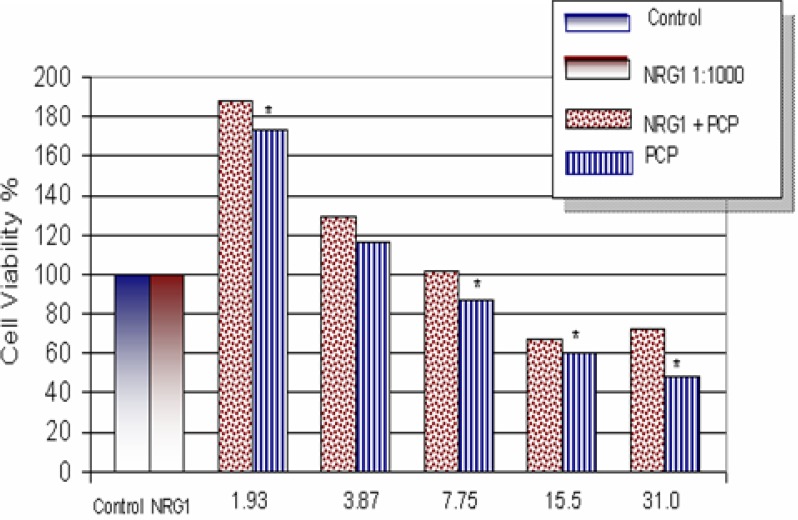
*Effect of NRG1-β on PCP toxicity in AML 12 Mouse Hepatocytes.* Cells were treated for 48hrs with PCP in the presence or absence of NRG1-β (0.01 nM). The number of metabolically active cells was determined by the MTT incorporation. The data are expressed as percentages of cell viability. Each point represents a mean value and standard deviation of three independent experiments (n = 3 independent experiments; 8 replications per treatment). *Significantly different (*p < 0.05*) from NRG1-β-treatment.

The effects of NRG1-β on PCP toxicity to AML 12 mouse hepatocytes are shown in Figure 2. Within the dose range of 0-31.0 µg/mL, a biphasic-response relationship was observed in both NRG1-β+PCP- and PCP-treated cells. The percentages for cell viability were recorded as 100.0 ± 0.0%, 188.0 ± 0.3%, 129.0 ± 0.3%, 102.0 ± 0.3%, 67.0 ± 0.2%, and 72.0 ± 0.1% at 0, 1.93, 3.87, 7.75, 15.5, and 31.0 µg/mL respectively for NRG1-β+PCP. Cell viability percentages for PCP treatments were recorded as 100 ± 0.0%, 173.0 ± 0.4%, 116.0 ± 0.4%, 87.0 ± 0.2%, 60.0 ± 0.2%, and 48.0 ± 0.1% at 0, 1.93, 3.87, 7.75, 15.5, and 31.0 μg/mL, respectively. The highest viability was achieved at 1.93 µg/mL. Cell viability percentages indicated that NRG1-β+PCP-treated cells had not been significantly impaired (except for the highest PCP concentration), while PCP-treated cells were appreciably reduced. Data obtained from this experiment strongly suggest that NRG1-β has the ability to rescue cell survival after PCP treatment by suppressing the toxic effects of PCP.

### Western Blot and Densitometric Analyses for c-fos Expression

In the studies herein, mouse hepatocytes were acutely exposed to PCP and NRG1-β+PCP treatments (0, 8, 16 μg/mL) and compared to the untreated (0) and 0.01 nM NRG1-β to determine the magnitude of changes in specific protein expressions. In each experiment, basal levels of specific protein expression were not detected in untreated and NRG1-β–treated cultures. Western-blot and densitometric analyses were performed according to the methodology section. The 62-kDa *c-fos* protooncogene is recognized as an immediate early gene and has been identified as a transcription factor that responds to DNA-damage [22]. We have previously reported that PCP has the ability to markedly induce a dose-dependent upregulation of the *c-fos* gene protein in HepG_2_ cells and primary catfish hepatocytes [18, 19, 23].

**Figure 3:**
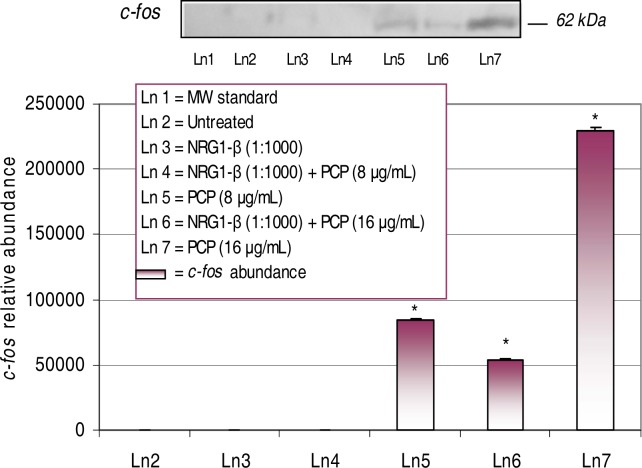
*Expression and relative abundance of the 62 kDa c-fos in AML 12 mouse hepatocytes exposed to PCP and NRG1-β + PCP for 48 h.* AML 12 mouse hepatocytes were treated with 8 µg/mL and 16 µg/mL concomitant treatments of PCP and NRG1-β + PCP. *c-fos* protein identification was assessed following exposure incubation period of 48 h. Inset shows a representative Wester n blot analysis. Each point represents a mean value and standard deviation of three experiments. *Significantly different (*p < 0.05*) from untreated (0 µg/mL PCP) and NRG1-β (1:1000) treated cells.

Western-blot and densitometric analyses of *c-fos* expression in PCP- and NRG1-β + PCP-treated AML 12 mouse hepatocytes for 48 h are shown in Figure 3 upon 48h of exposure, a dose-dependent upregulation of the *c-fos* protein was observed in concomitant treatments of PCP and NRG1-β+PCP. The magnitude of *c-fos* expression was highly correlated with increased levels of PCP toxicity. For example, densitometric analysis showed a significant (*p*<0.05) increase of *c-fos* relative abundance of 83,714 at 8 µg PCP/mL, 54,115 at 16 µg NRG1-β+PCP/mL, and 229,374 at 16 µg PCP/mL. We did not detect the *c-fos* protein at 8 μg NRG1-β+PCP/mL; suggesting that NRG1-β attenuates PCP-stimulated *c-fos* expression at low levels of PCP-toxicity.

### Western Blot and Densitometric Analyses for HSP70 Expression

HSP70, a member of the heat shock protein family, is a highly conservative molecular chaperone with strategic functions that respond to conditions of environmental stress, including tissue damage, inflammation, and mutant proteins associated with genetic abnormalities [24]. We have previously reported that the overexpression of the HSP70 gene protein is a dose-dependent event in HepG_2_ cells exposed to PCP [18, 23].

**Figure 4:**
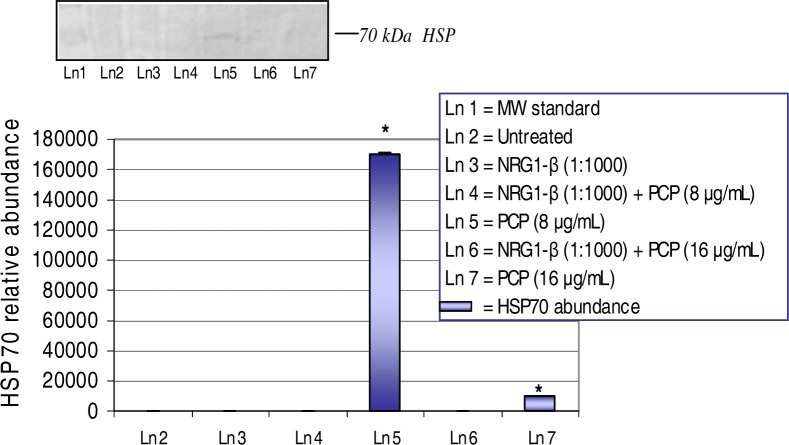
*Expression and relative abundance of the 70 kDa heat shock (HSP70) in AML 12 mouse hepatocytes exposed to PCP and NRG1-β + PCP for 48 h.* AML 12 mouse hepatocytes were treated with 8 μg/mL and 16 μg/mL concomitant treatments of PCP and NRG1-β+PCP. HSP70 protein identification was assessed following exposure incubation period of 48 h. Inset shows a representative Western blot analysis. Each point represents a mean value and standard deviation of three experiments. *Significantly different (*p<0.05*) from untreated (0 µg/mL PCP) and NRG1-β (1:1000) treated cells.

The expression and relative abundance of the 70-kDa heat shock protein in AML 12 mouse hepatocytes exposed to PCP and NRG1-β+PCP treatments for 48 h are shown in Figure 4. A dose-dependent upregulation of the HSP70 gene protein was demonstrated in both treatments of PCP and NRG1-β+PCP at 48 h. PCP-toxicity induced the upregulation of HSP70 at 8 μg PCP/mL with a relative abundance of 170,584. However, a drastic decrease (*p*<0.05) or down-regulation of HSP70 expression was demonstrated at 16 μg PCP/mL with a relative abundance of 10,000. NRG1-β completely suppressed the expression of HSP70 at 8 µg NRG1-β+PCP/mL and 16 µg NRG1-β+PCP/mL. These results suggest that NRG1-β has the ability to rescue cells from the consequences of proteolytic activity induced by PCP-toxicity.

### Western-Blot and Densitometric Analyses for GADD153 Expression

**Figure 5:**
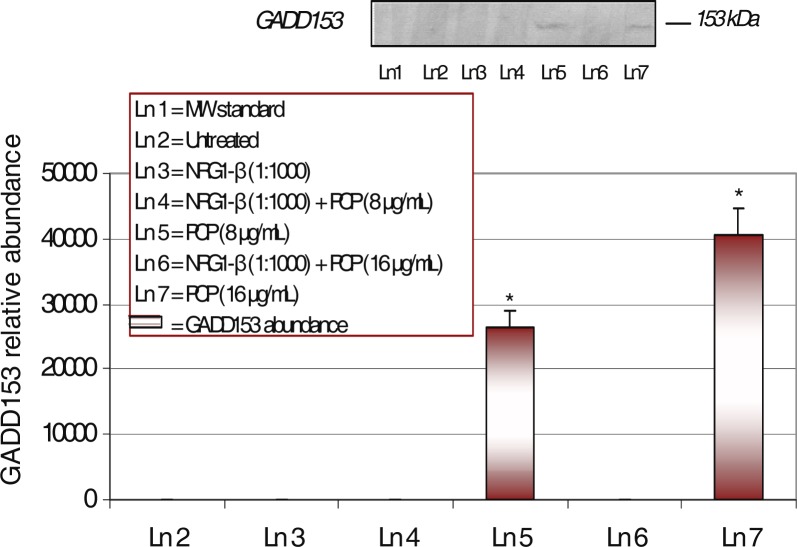
*Expression and relative abundance of the 153 kDa GADD in AML 12 mouse hepatocytes exposed to PCP and NRG1-β + PCP for 48 h.* AML 12 mouse hepatocytes were treated with 8 μg/mL and 16 μg/mL concomitant treatments of PCP and NRG1-β + PCP. GADD153 protein identification was assessed following exposure incubation period of 48 h. Inset shows representative Wester n blot analysis. Each point represents a mean value and standard deviation of three experiments. *Significantly different (*p ≤ 0.05*) from untreated (0 µg/mL PCP) and NRG1-β (1:1000) treated cells.

The GADD153 gene protein is robustly induced by genotoxic stress and is coordinately regulated with the endoplasmic reticulum (ER) [25]. The expression and relative abundance of the 153-kDa GADD in AML 12 mouse hepatocytes exposed to PCP and NRG1-β+PCP treatments for 48 h are shown in Figure 5.

We have previously reported that PCP can induce a dose-dependent expression of the GADD153 gene protein in HepG_2_ cells [18]. In this experiment, stimulated cells resulted in a dose-dependent (*p*<0.05) upregulation of the GADD153 protein with relative abundances of 26,411 and 40,626 respectively for 8 μg PCP/mL and 16 μg PCP/mL. Conversely, the expression of GADD153 was completely repressed at 8 µg NRG1-β+PCP/mL and 16 µg NRG1-β+PCP/mL. Data obtained from this experiment indicated that NRG1-β has the ability to attenuate growth arrest and DNA-damage activity at low and high levels of PCP-toxicity.

### Western-Blot and Densitometric Analyses for p53 Expression

**Figure 6:**
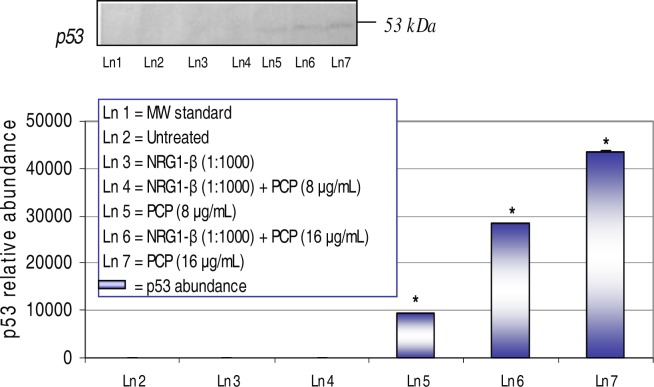
*Expression and relative abundance of p53 in AML 12 mouse hepatocytes exposed to PCP and NRG1-β+ PCP for 48 h*. p53 protein identification was assessed following exposure incubation period of 48 h. Inset shows representative Western blot analysis. Each point represents a mean value and standard deviation of three independent experiments. *Significantly different (*p < 0.05*) from untreated (0 μg/mL) and NRG1-β (1:1000) treated cells.

The 53-kDa p53 gene protein, also known as the tumor suppressor gene, senses DNA damage and responds by arresting the cell cycle [26]. Western-blot and densitometric analyses of the p53 gene protein in AML 12 mouse hepatocytes exposed to PCP and NRG1-β + PCP for 48 h are shown in Figure 6. We have previously reported that PCP has the ability to transcriptionally activate the p53 tumor suppressor gene in HepG_2_ cells [18, 23]. NRG1-β has the ability to repress the p53 protein at lower levels of PCP-toxicity. We did not detect the p53 expression at 8 µg NRG1-β+PCP/mL. However, we observed a dose-dependent increase (*p*<0.05) upregulation of the p53 protein with relative abundances of 9,502, 28,339 and 43,458 respectively for 8 µg PCP/mL, 16 µg NRG1-β+PCP/mL, and 16 µg PCP/mL; demonstrating the incompetence of NRG1-β to suppress DNA damage at higher levels of PCP toxicity.

### Western-Blot and Densitometric Analyses for Cyclin D1 and Caspase-3 Expression

**Figure 7:**
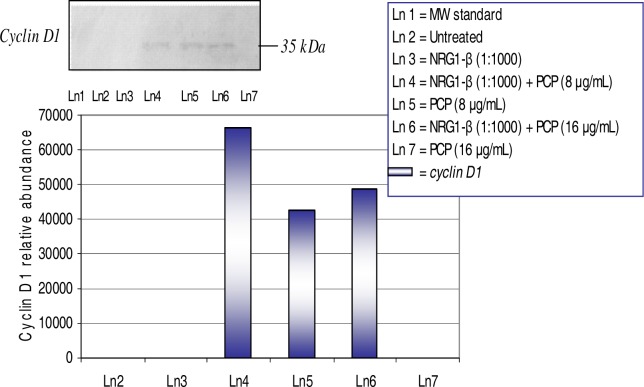
*Expression and relative abundance of cyclin D1 in AML 12 mouse hepatocytes exposed to PCP and NRG1-β + PCP for 48h.* Cyclin D1 protein identification was assessed following exposure incubation period of 48 h. Inset shows a representative Western-blot analysis. The following values were compared to untreated (0 µg/mL) and NRG1-β (1:1000) treated cells.

The overexpression of cyclin D1 in hepatocytes is indicative of G_1_/S transition and mitogenic response [27]. Expression and relative abundance of the 35–kDa cyclin D1 in AML 12 mouse hepatocytes exposed to PCP and NRG1-β + PCP treatments for 48 h are shown in Figure 7. We have previously reported that PCP has the ability to elicit a mitogenic response in HepG_2_ cells, primary catfish hepatocytes, and AML 12 mouse hepatocytes [19, 20, 23]. In this experiment, we observed a dose-dependent overexpression of cyclin D1 with relative abundances of 66,371 at 8 μg NRG1-β+PCP/mL, 42,764 at 8 µg PCP/mL, and 48,757 at 16 µg NRG1-β+PCP/mL. This finding supports the fact that NRG1-β has the ability to protect hepatocytes in the presence of PCP.

**Figure 8:**
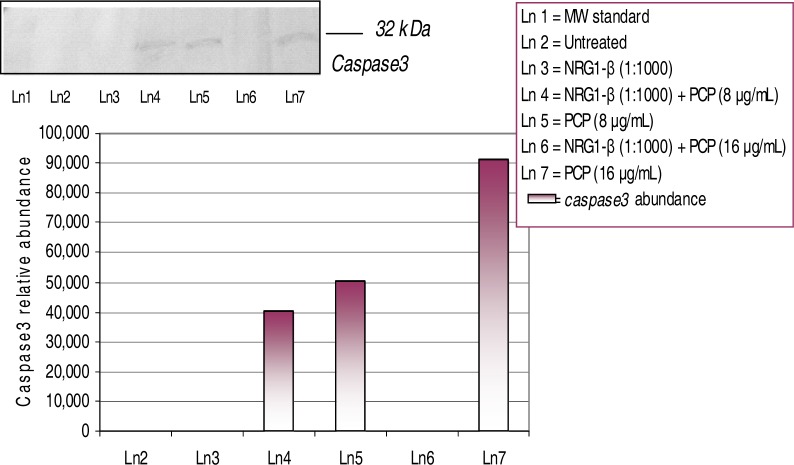
*Expression and relative abundance of caspase-3 in AML 12 mouse hepatocytes exposed to PCP and NRG1-β + PCP for 48h.* Caspase-3 protein identification was assessed following exposure incubation period of 48 h. Inset shows a representative Western-blot analysis. The following values were compared to untreated (0 µg/mL) and NRG1-β (1:1000) treated cells.

The upregulation of caspase-3 expression is strongly associated with the readiness of cells to undergo apoptosis [28]. The expression and relative abundance of the 32-kDa caspase-3 protein in AML 12 mouse hepatocytes exposed to PCP and NRG1-β + PCP treatments for 48 h are shown in Figure 8. In this experiment we observed a dose- dependent activation of the caspase-3 protein with relative abundances of 40, 223 at 8 µg NRG1-β+PCP/mL, 50,160 at 8 µg PCP/mL, and 91,239 at 16 µg PCP/mL. In the presence of NRG1-β, the caspase-3 protein was repressed or down-regulated at 16 µg NRG1-β+PCP/mL; demonstrating a protective effect of NRG1-β at higher levels of PCP exposure. Here, we clearly demonstrate the ability of NRG1- β to block programmed cell death in PCP-treated mouse hepatocytes.

## Discussion

### Neuregulin 1-βeta-Induced Protective Effects

The present study was designed to investigate the protective effects of NRG1- β on PCP-induced cytotoxicity in AML 12 mouse hepatocytes. To our knowledge, the effects of NRG1-β treatment on hepatocyte survival have not been previously reported. We have previously reported that PCP is acutely toxic and causes cell injury to AML 12 mouse hepatocytes [20]. In that study, the 48 h-LC_50_ was computed to be 16.0 ± 2.0 μg PCP/mL. In the present study, we observed a similar biphasic response pattern with respect to cell viability. Our *in vitro* cell viability studies indicate that NRG1-β has a direct cytoprotective effect on hepatocytes against PCP-toxicity.

The members of the NRG family are produced by either neuronal or mesenchymal cells, and mediate their effects by binding to and signaling by the ErbB family of receptors [2, 9]. However, we provide clear evidence that NRG1-β was found to display a protective effect on AML 12 mouse hepatocytes caused by PCP toxicity (Figure 2). All NRG proteins contain an extracellular EGF-like domain, which is essential for bioactivity [29]. In a previous study, NRG1-β treatment of neonatal rat ventricular myocytes was shown to inhibit daunorubicin-induced apoptosis and the activation of caspase-3 [30]. A similar investigation demonstrated the survival effects of NRG1-β on norepinephrine cytotoxicity in adult rat ventricular myocytes by suppressing beta-adrenergic receptor-stimulated apoptosis [31]. In the present study, we have demonstrated that NRG1-β has to ability to inhibit PCP-induced apoptosis by completely suppressing the activation of caspase-3 at higher levels of PCP toxicity (Figure 8).

NRGs and their receptors can influence a network of survival signaling pathways, which is likely to vary in many cell types. The phosphoinositide 3-kinase (PIK3), which is specifically recruited by ErbB-3 and the ErbB-1T3 chimeric receptor, is a well-known regulator of cell growth and survival [32]. Abundant evidence has shown that the protective effects of NRGs are mediated by the PI3K signaling pathway [33, 34, 35]. In a very recent study, NRG induced a significant protective effect from β-amyloid 25–35 peptide-induced cell death [33]. The same study revealed that NRG treatment produced elevation in the levels of the antiapoptotic protein BclxL. The NRG-mediated BclxL elevation was regulated by protein kinase C (PKC). Results from that study suggested that NRG might affect cell viability by using two signaling pathways: activation of PI3K/PKB/AkT pathway and activation of PKC, which resulted in increasing levels of the antiapoptotic protein BclxL [33]. More specifically, NRG1-β has been shown to have a prosurvival effect on cardiac myocytes via the PI3K/Akt pathway [34]. The NRG-β isoform is a potent Schwann cell survival factor that binds to and activates a heterodimeric ErbB2/ErbB3 receptor complex [35]. Moreover, corroboration that NRG is mediated by the PIK3 pathway was demonstrated when NRG rapidly signaled the phosphorylation of mitogen-activated-protein kinase (MAPK) and the serine/threonine kinase Akt in serum-starved Schwann cells [35]. The same study used PIK3 inhibitors that blocked the NRG-mediated rescue of Schwann cells, as well as Akt, MAPK, and Bad; demonstrating the involvement of the PIK3 pathway [35].

Another possible mechanism of NRG1-β against PCP-toxicity in mouse hepatocytes is the involvement of the MAPK pathway. The MAPK pathway is thought to be directly responsible for regulating cell proliferation, differentiation, and survival. A number of investigations have implicated the MAPK pathway in NRG-β mediated survival [35, 36, 37]. A recent study demonstrated that NRG1-induced activation of ErbB4 stimulates the MAPK, PI3K, and cyclin-dependent kinase-5 (cdk5) pathways in cultured rat cerebellar granule neurons [37]. MAPK is a known downstream effector of the ErbB receptors. Therefore, in the present study, it is also possible that NRG1-β may exert its protective activity in PCP-treated mouse hepatocytes via the MAPK pathway.

Supporting evidence suggests that ErbB3 requires the association of other ErbB receptors or the EGFr to form an active signaling complex in hepatocytes [38]. Although NRGs are not primary mitogens in rat liver, they could regulate differentiation during development, maintenance of differentiated functions during regeneration, or metabolism in response to nutritional status [38]. A previous study demonstrated that a recombinant peptide corresponding to the EGF domain of the ß-1 isoform of heregulin (NRG1-β) bound to rat hepatocytes via the ErbB3 receptor, induced receptor phosphorylation, and stimulated DNA synthesis [38]. A similar investigation has shown that during rat liver development and regeneration, diverse ErbB receptor proteins are expressed [39]. In the present study, cell survival and cytoprotective effects of NRG1-β were assessed by the MTT- cell respiration assay. Cell viability data indicated that NRG1-β possesses a potent protective effect against PCP-induced cytotoxicity in AML 12 mouse hepatocytes (Figure 2). We propose that NRG1-β protective effects in PCP-treated hepatocytes are orchestrated by binding to ErbB receptors, and in turn the PI3K or MAPK pathway is solicited.

### NRG1-β Effects on PCP-Induced c-fos Expression

Gene expression is controlled and regulated by many transcription factors in order for the cell to adjust to environmental or genetic modifications. We present evidence that NRG1-β has the ability to attenuate stress-related events as a consequence of PCP-toxicity. Specifically, we show that PCP induces stress-related gene expression and death of AML12 mouse hepatocytes, where NRG1-β protected them from PCP-induced death. To assess the cellular injury response in AML 12 mouse hepatocytes, we examined the effect of NRG1-β on the expression of stress-related gene proteins as a consequence of PCP-toxicity. In this study, we report that an appreciable dose-dependent expression of the *c-fos* protein was observed after 48 h of exposure to concomitant treatments of PCP and NRG1-β+PCP (Figure 3, Table 1).

The transcriptional activation of immediate early transcription factors such as *c-fos* is thought to be essential for mitogen-induced progression through the cell [40]. *c-fos* is a constituent of the immediate early transcription factor activator protein 1 (AP-1) heterodimeric complex and has been implicated as an positive modulator of G_1_-to-S-phase progression and cell proliferation [40, 41, 42]. The induction of the *c*-*fos* gene involves both transcriptional and post-transcriptional machinery [43]. Once stimulated, *c-fos* conjoins with c*-jun*, a transcription factor of the Jun family, and forms the heterodimeric complex, AP-1 [43]. Moreover, oxidative stress and DNA damage can stimulate c-*fos* expression and thus increase AP-1 transcription factor activity [44, 45]. The *c-fos* protooncogene plays a vital role in mitogenesis by inducing the expression of genes necessary for the activation of G1 cyclins [46]. For example, cyclin D1 mRNA is increased by the upregulation of *c-fos* [47] and mitosis-and mitogen-stimulated cyclin D1 transcription are repressed in cells deficient for *c-fos* and FosB [46]. It is well documented that mitogen-stimulated cyclin D1 requires the PIK3 activity [48, 49] and upon activation, PI3K stimulates *c-fos* transcription [50]. We have previously reported that the potential toxicity of PCP transcriptionally activates the *c-fos* gene protein in HepG_2_ cells and in primary catfish hepatocytes [18, 19, 23]. In the present study, we observed a dose-dependent upregulation of the *c-fos* protein in PCP-treated mouse hepatocytes (Figure 3, Table 1). This finding is consistent with our previous results and demonstrates similar PCP mechanistic activity across cell lines. We also observed a NRG1-β down regulation or repression of *c-fos* expression; indicating that NRG1-β has the ability to attenuate the activation of *c-fos* expression in PCP-treated AML mouse hepatocytes.

### NRG1-β Effect on PCP-Induced HSP70 Expression

The 70-kDa HSP gene, a member of the heat shock protein molecular chaperone family, is involved in protein folding, translocation, and refolding of intermediates and proteases, while ensuring the efficient degradation of damaged and short-lived proteins [51]. Under stressful conditions, the accumulation of unfolded proteins in the ER leads to the induction of transcriptionally activated genes that encode molecular chaperones and folding enzymes [52]. Upon a variety of stress stimuli, signals are transduced from the ER to the cytoplasm and the nucleus to eventually result in adaptation for survival or induction of apoptosis [53]. HSP70 is documented as a general anti-apoptosis protein where it protects cells from the consequences of proteolysis by caspase-3-like proteases [54]. Moreover, the HSP70 gene protein is highly associated with the inflammatory response in lung epithelium and myocardial damage [55, 56]. We have previously shown that PCP can potently activate the HSP70 protein in HepG_2_ cells [18, 23]. In the present study, a dose-dependent increased expression of HSP70 was induced as a result of PCP-toxicity (Figure 4, Table 1). These results may be directly linked to the phenomenon in which the HSP70 gene enhances a cell signaling cascade that initiate protein repair. In the presence of NRG1-β, we observed a down regulation or inhibition of HSP70 expression in PCP-treated cells at 48 h of exposure. Our results are supported by a recent study that demonstrated the ability of NRG1-β to reverse inflammation and oxidative stress-related genes in focal ischemia of the rat brain and in a rat neuroblastoma cell line [13]. Here, we report that NRG1-β has the ability to attenuate proteolytic activity as a consequence of PCP-toxicity.

### NRG1-β Effect on PCP-Induced GADD153 Expression

The GADD153 gene protein is a CCAAT/enhancer-binding protein (C/EBP)-related gene whose expression is induced in response to growth arrest and DNA damage [57]. The mechanism responsible for the activation of GADD153 expression after DNA damage remains unclear. GADD153 is induced by DNA- and cellular damage in a dose-dependent manner [58, 59]. The magnitude of GADD153 expression is proportional to the extent of cellular injury with maximal GADD153 promoter activity occurring under circumstances of severe toxicity to the cell [58]. Several investigations have reported that the induction of GADD153 is strongly correlated with the onset of apoptosis [60, 61]. Moreover, it has been demonstrated that elevated GADD153 expression depletes cells of essential thiols and down-regulates Bcl2 expression by inhibiting *bcl2* transcription; thus sensitizing cells for death [58]. GADD153 is a potent mediator of the p53 gene protein in response to a variety of DNA damaging agents, inducing directly or indirectly G1 arrest and/or apoptosis [62]. We have previously reported that PCP-toxicity can induce a dose-dependent GADD153 expression in HepG_2_ cells [18]. In the present study, we also observed a dose-dependent elevated expression of the 153-kDa GADD protein in PCP-treated hepatocytes at 48 h of exposure (Figure 5, Table 1). Consistent with our previous findings, NRG1-β down-regulated or completely repressed the GADD153 expression in PCP-treated hepatocytes. In this study, the GADD153 expression is consistent with a previous study that demonstrated the ability of NRG1-β to reverse stress-related gene expression [13].

### NRG1-β Effect on PCP-Induced on p53 Expression

The p53 gene is a tumor suppressor protein that plays a regulatory role in cell cycle control and apoptosis. p53 is a checkpoint molecule in G_1_ arrest caused by DNA damage and is closely dependent on transcriptional activation of the CKI *p21* target gene [63]. Three major events associated with the p53 tumor suppressor gene are growth arrest, DNA repair, and apoptosis. Moreover, the ability of p53 to function as a sequence-specific DNA-binding protein appears to be essential to the function of p53 as a tumor suppressor [64, 65]. It has been reported that increased p53 expression is directly linked to missense mutations, which result in loss of its transcriptional activator function [66]. Our laboratory has previously reported that PCP has the ability to induce a dose-dependent expression of the 53-kDa p53 protein in HepG_2_ cells [18, 23]. In the present study, NRG1-β attenuated the expression of p53 at lower levels of PCP; however, PCP-treated hepatocytes demonstrated an increase in p53 expression (Figure 6, Table 1). In support of these results, it has been documented that in response to DNA damage, cells facilitate a rapid increase in wild-type p53 levels and a temporarily G1 arrest; allowing time for DNA to be repaired before being copied [67]. When optimal repair after DNA damage is irreparable, p53 initiates the signal to promote apoptosis [68].

### NRG1-β Effect on PCP-Induced cyclin D1

Cell cycle transition from G_1_ to S phase is tightly regulated by distinct cyclin-dependent kinases (cdks) which are instrumental in cell cycle progression [69]. Cyclin D/Cdk4 complexes phosphorylate the retinoblastoma gene product early in the G_1_ phase of the cell cycle [69]. The 35-kDa cyclin D1 is overexpressed in several types of carcinomas and, therefore, suggested to play an important role in tumorigenesis and tumor progression including hepatocellular carcinoma [70, 71, 72]. When cyclin D1 is upregulated due to gene amplification, gene rearrangement, protein stabilization or other mechanisms, cyclin D1 acts as an oncogene by intensifying cell transformation, either alone [73] or in combination with activated *ras* [74], thereby shortening the G_1_ phase of the cell cycle. Interestingly, the induction of apoptosis is closely associated with an increase in cyclin D1-dependent kinase activity [75, 76, 77]. Notably, alterations in cyclin D1/Cdk4 caused by inhibition of ErbB2 support a critically role for cyclin D1 in ErbB2-mediated cell cycle progression [78]. Primary hepatocytes in culture readily proliferate in response to mitogens such as EGF [79]. Previous studies have shown that cyclin D1 is up-regulated during hepatocyte proliferation in culture [27, 80, 81]. We have previously demonstrated that PCP is capable of provoking a mitogenic response in HepG_2_ cells, primary catfish hepatocytes, and AML 12 mouse hepatocytes [19, 20, 23]. Results from the present study showed a dose-dependent activation of the 35-kDa cyclin D1 protein in PCP-treated cell in the presence of NRG1-β (Figure 7). However, in the absence of NRG1-β, PCP-treated cells demonstrated a down-regulated or repressed expression of the cyclin D1 protein; probably due to cell death at high levels of PCP- toxicity. These results are consistent with previous data in this study that demonstrate the protective effects of NRG1-β against PCP-toxicity.

### NRG1-β Effects on PCP-induced Caspase-3 Expression

Caspases are the major enforcers of cell death, serving as molecular executioners to destroy many proteins required for maintenance of cellular homeostasis [82]. Caspase-3 is a major modulator of apoptotic activity. Apoptotic inhibitors, such as Bcl-2 and NF-kappa B, play a crucial role in the mechanism of anti-apoptosis of tumors [83, 84]. Caspase-3 is highly associated with apoptosis and is only cleaved and activated once the process of apoptosis is irreversible [85, 86]. Interestingly, it has been documented that PKC activity precedes the activation of caspase-3 [87]. A recent study demonstrated that the overexpression of PKC resulted in an increase of apoptosis, whereas its inhibition blocked caspase-3 activity and decreased apoptosis [87]. We have previously demonstrated that PCP causes cell injury and is cytotoxic to HepG_2_, primary catfish hepatocytes, and AML 12 mouse hepatocytes [18, 19, 20, 23]. In this study, we clearly demonstrate the ability of PCP to induce apoptosis in AML 12 mouse hepatocytes. This event was demonstrated by the upregulation of the 32-kDa caspase-3 protein in PCP-treated hepatocytes (Figure 8). Moreover, the caspase-3 protein was down-regulated or repressed in the presence of NRG1-β at higher levels of PCP. These results demonstrate the ability NRG1-β can exert an anti-apoptotic effect in PCP–treated hepatocytes. The mechanism by which NRG1-β attenuates caspase-3 expression is not clear. Future studies from our laboratory will investigate whether NRG1-β regulates pro-apoptotic gene expression by interfering with various transcriptional signaling pathways.

## Conclusions

We have demonstrated that NRG1-β plays a cytoprotective role in AML 12 mouse hepatocytes exposed to PCP. NRG1-β was able to protect AML 12 mouse hepatocytes from cell injury by suppressing the toxic effects of PCP. NRG1-β has the ability to attenuate stress-related gene expression in PCP-treated AML 12 mouse hepatocytes. Western-blot analysis strongly indicated that PCP has the ability to cause oxidative stress and inflammatory reaction (*c-fos*), growth arrest and DNA damage (GADD153), proteotoxic effects (HSP70), and cell cycle arrest as consequence of DNA damage (p53). A mitogenic response was demonstrated by the upregulation of the 35-kDa cyclin D1 protein in PCP-treated hepatocytes. PCP-induced apoptosis was demonstrated by the overexpression of the 32-kDa caspase-3 protein. PCP-induced toxicity was attenuated or reversed in the presence of NRG1-β. To our knowledge, the anti-inflammatory activity of NRG1-β, is a novel finding and could represent efficient treatment for hepatic inflammatory disorders. Future studies from our laboratory will investigate the regulatory activity of NRG1- β on pro-inflammatory gene expression.

## Figures and Tables

**TABLE 1:** Relative abundance of significantly up-regulated proteins in AML 12 mouse hepatocytes exposed for 48 hrs to PCP in the absence or presence of NRG1-β (0.01 nM).

*Gene Protein*	*Biological Function / Response*	*NRG1+PCP 8μg/mL*	*PCP 8μg/mL*	*NRG1+PCP 16μg/mL*	*PCP 16μg/mL*
*c-fos*	AP-1 component Signaling-immediate early gene	-	83,714	54,115	229,374
	DNA-damage Molecular chaperone				
HSP70	Oxidative stress Protein alterations	-	170,584	-	10,000
	Cell cycle regulator				
GADD153	DNA repair	-	26,441	-	40,626
	DNA-damage				
p53	Cell cycle- regulator Apoptosis	-	9,502	28,389	43,458
	DNA-damage				
